# Clinical efficacy and safety of icaritin in patients with hepatocellular carcinoma: a real-world study

**DOI:** 10.3389/fphar.2026.1677794

**Published:** 2026-02-12

**Authors:** Jingchang He, Lanlan Zhuang, Yi Lei, Biao Wang, Huaiyu Chen, Shuai Kang, Dingli Liu, Kunyuan Wang, Wenxuan Yu, Yuchen Lu, Yang Cheng, Yun Zhu

**Affiliations:** 1 Department of Infectious Diseases, Nanfang Hospital, Guangdong Provincial Key Laboratory for Prevention and Control of Major Liver Diseases, Southern Medical University, Guangzhou, China; 2 Digestive Department, Guangzhou Women and Children’s Medical Center, Guangzhou Medical University, Guangzhou, China; 3 Department of Hepatobiliary Surgery, Nanfang Hospital, Southern Medical University, Guangzhou, China; 4 School of Traditional Chinese Medicine, Southern Medical University, Guangzhou, China

**Keywords:** efficacy, HRQOL, icaritin, safety, uHCC

## Abstract

**Background:**

Icaritin, a natural compound extracted from Epimedium, has demonstrated efficacy and a favorable safety profile in treating hepatocellular carcinoma (HCC), providing an option for patients intolerant to conventional treatment. However, real-world data remain limited. To assess the therapeutic potential and safety of icaritin for patients with unresectable HCC (uHCC), we conducted a retrospective analysis at our center.

**Methods:**

This study analyzed 26 HCC patients treated with icaritin-based regimens (monotherapy, n = 10; combination therapy, n = 16) at Nanfang Hospital (June 2018–June 2024). We evaluated efficacy outcomes, including objective response rate (ORR), disease control rate (DCR), progression-free survival (PFS), and overall survival (OS), as well as safety and health-related quality of life (HRQOL).

**Results:**

The monotherapy group showed a DCR of 60.0%, median PFS of 3.5 months, and a median OS of 9.4 months. The combination therapy group had a DCR of 93.8%, a median PFS of 5.7 months, and a median OS of 10.6 months. The incidence of Grade 3 treatment-related adverse events (TRAEs) was 13.9%, and no Grade 4+ TRAEs were observed. HRQOL was maintained throughout treatment in either group. One patient with BCLC stage C achieved a partial response after 2 months of icaritin monotherapy and a complete response after 8 months, with PFS exceeding 18 months.

**Conclusion:**

Icaritin-based therapy has certain efficacy and a favorable safety in uHCC, suggesting a therapeutic alternative for uHCC patients ineligible for standard treatments.

## 1 Introduction

Hepatocellular carcinoma (HCC), representing 75%–85% of primary liver cancer cases, is a major global health challenge with high mortality (Sung et al., 2021). Approximately half of all HCC patients worldwide are located in China (Llovet et al., 2021; Han et al., 2024). The disease is often diagnosed at advanced stages when curative surgery is no longer feasible, contributing to a 5-year survival rate of only 12.1% in China, underscoring the critical need for better treatment options (Nationat Health Commission of the People’s Republic of China, 2024).

Guidelines recommend systemic antitumor therapy and/or locoregional therapy for patients with unresectable HCC (uHCC) (Nationat Health Commission of the People’s Republic of China, 2024). While first-line targeted agents such as lenvatinib and sorafenib improve survival, the median overall survival (OS) remains limited to 9–14 months (Kudo et al., 2018; Finn et al., 2020). Recent advances in immune checkpoint inhibitors (ICIs) have gained prominence in systemic therapy. The IMbrave150 trial reported that bevacizumab plus atezolizumab achieved a median OS of 19.2 months (Cheng et al., 2022). The HIMALAYA study reported a 5-year OS rate of 19.6% with tremelimumab plus durvalumab (Rimassa et al., 2024).

HCC develops in a unique immunological environment characterized by immune system dysregulation, chronic inflammation, and frequent autoimmune-like phenomena, which contributes to the fact that some patients already exhibit significant liver impairment at diagnosis (Aravalli, 2013). Approximately 20% of HCC patients present with impaired liver function, rendering them ineligible for standard systemic treatments. This population has limited therapeutic options, resulting in poor prognoses and severely compromised quality of life (Park et al., 2015; Allemani et al., 2018). Moreover, anticancer agents commonly used in HCC—such as targeted and immunotherapy—can cause direct hepatotoxicity or immune-related liver injury, leading to treatment discontinuation.

Phytochemical agents, such as icaritin, have shown potential in the treatment of HCC patients with compromised liver function. Icaritin, a prenylflavonoid derived from Epimedium, exhibits anti-inflammatory and immunomodulatory properties. Icaritin has demonstrated potent pharmacological activity in multiple *in vitro* and *in vivo* studies, primarily through mechanisms related to apoptosis. Pharmacokinetic studies suggest that icaritin undergoes diverse metabolic pathways, including hydration, hydroxylation, dehydrogenation, glycosylation, and glucuronidation (Huong and Son, 2023). Preclinical studies suggest that icaritin exerts antitumor effects through multiple mechanisms, including induction of apoptosis, cell cycle regulation, inhibition of angiogenesis and metastasis, and modulation of the tumor microenvironment (Zhang et al., 2020; Tan et al., 2016; Li et al., 2021; Bailly, 2020). These mechanisms highlight its potential as a novel therapeutic option for uHCC.

A phase III clinical trial has demonstrated favorable biosafety and OS benefits with icaritin in advanced HCC patients (Sun et al., 2021). Accordingly, the 2024 Chinese Society of Clinical Oncology (CSCO) guidelines recommended icaritin as a Class 1 treatment (level 1B evidence) for patients with advanced HCC and hepatic impairment (Chinese Society of Clinical Oncology, 2024). A real-world study presented at the 2024 American Society of Clinical Oncology (ASCO) annual meeting also reported that icaritin combined with targeted, immune, or interventional therapy achieved a median progression-free survival (PFS) of 8.93 months with good tolerability and safety (Shao et al., 2024).

Despite these promising findings, clinical reports on the use of icaritin in HCC remain limited. This study assessed the clinical efficacy and safety of icaritin in the comprehensive treatment of patients with HCC through a case series study analysis. This study aimed to provide a clinical basis for establishing new treatment paradigms for HCC.

## 2 Methods

### 2.1 Participants

This study included HCC patients who received icaritin-based treatment at Nanfang Hospital, Southern Medical University, between June 2018 and June 2024. The study included patients aged 18 years or older with HCC confirmed either clinically or pathologically. Eligible patients were required to have at least one measurable lesion on contrast-enhanced computed tomography (CT) or magnetic resonance imaging, and to have completed at least one treatment cycle to enable efficacy assessment.

The study population consisted of HCC patients who were deemed ineligible for standard therapies due to treatment intolerance, resistance, or poor baseline conditions (e.g., impaired liver function). Icaritin was administered orally at a standard dose of 600 mg twice daily; a reduced dose of 300 mg twice daily was used in some patients based on individual tolerability. Doses were taken with warm water 30 min after breakfast and dinner. In the combination therapy group, icaritin was used alongside targeted agents (lenvatinib or anlotinib), ICIs (tislelizumab or candonilimab), or transarterial chemoembolization (TACE). Patients were followed up until 1 October 2024.

### 2.2 Clinical data collection and outcome definitions

Blood counts, coagulation function, renal function, liver function, and alpha-fetoprotein (AFP) levels were collected. Efficacy was assessed independently by two radiologists, and a consensus was reached through discussion if there was a difference in opinion. Tumor response was estimated according to the modified RECIST (mRECIST) criteria, which were classified as (i) complete response (CR), (ii) partial response (PR), (iii) stable disease (SD), or (iv) progressive disease (PD).

OS, PFS, the objective response rate (ORR), the disease control rate (DCR), time to progression (TTP), and safety were evaluated in both groups after icaritin treatment. TRAEs were graded according to the Common Terminology Criteria for Adverse Events version 5.0 (CTCAE v5.0). Health-related quality of life (HRQOL) was evaluated using the European Organization for Research and Treatment of Cancer Quality of Life Questionnaire for Hepatocellular Carcinoma (EORTC QLQ-HCC18) (Li et al., 2017; Serrano et al., 2022). The scale covers eight symptom domains, including fatigue, body pain, appetite loss, abdominal swelling, jaundice, skin problems, fever, and sexual problems. Scoring was performed via a 4-point Likert scale (1–4), with higher scores indicating poorer quality of life. The score was evaluated at baseline and every 3 months after initiating icaritin therapy.

### 2.3 Statistical analyses

Statistical analyses were performed using SPSS software (version 27.0). Survival analysis was analyzed using the Kaplan-Meier method. PFS was defined as the time from the initiation of icaritin administration to disease progression or death from any cause, whereas OS was defined as the time from icaritin administration to death. Categorical variables were compared using Fisher’s exact test or the Chi-square test, as appropriate. Univariate Cox regression was used to identify potential prognostic factors.

## 3 Results

### 3.1 Patient characteristics

**FIGURE 1:**
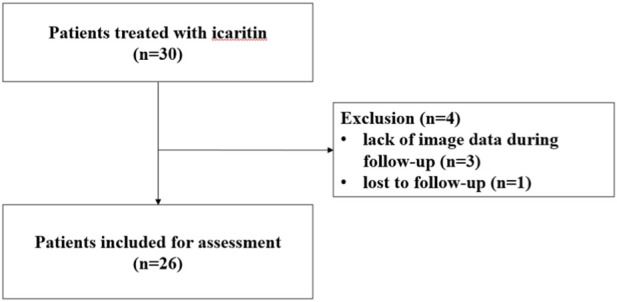
Flowchart of patient selection in the study.

Of 30 uHCC patients treated with icaritin, 4 were excluded (3 lacked imaging data, and 1 was lost to follow-up), leaving 26 patients included for assessment (Figure 1). There was a male predominance (22 males, 84.6%; 4 females, 15.4%) and a median age of 53 years (range: 21–75). Ten patients received icaritin monotherapy, mainly due to impaired liver function or intolerance to standard systemic therapies, whereas 16 patients received icaritin combined with local or systemic therapy, often as a subsequent-line option after prior treatment failure. No statistically significant differences in baseline characteristics were observed between the monotherapy and combination groups. Based on the Child‒Pugh classification, 10 patients (38.5%) were graded as A, 13 (50.0%) as B, and 3 (11.5%) as C. According to the Barcelona Clinic Liver Cancer (BCLC) staging system, the majority of patients were classified as stage C (76.9%), whereas 19.2% were in stage D. Among the combination therapy patients, treatment regimens included targeted–immunotherapy (n = 9), targeted agents alone (n = 2), immunotherapy alone (n = 3), TACE (n = 1), and radiotherapy (n = 1). The median duration of follow-up was 32.5 months (Table 1).

**TABLE 1:** Clinical characteristics of the patients.

Characteristics	Icaritin monotherapy (N = 10)	Icaritin-based combination therapy (N = 16)	Total (N = 26)	P value
Male, no. (%)	9 (90.0)	13 (81.3)	22 (84.6)	0.496
Age in years, median (range)	55 (44–75)	49 (21–75)	53 (21–75)	0.42
ECOG-PS, no. (%)	​	​	​	0.534
0	4 (40.0)	4 (25.0)	8 (30.8)	​
1–2	4 (40.0)	10 (62.5)	14 (53.8)	​
3	2 (20.0)	2 (12.5)	4 (15.4)	​
Child-Pugh classification, no. (%)	​	​	​	0.053
A	6 (60.0)	4 (25.0)	10 (38.5)	​
B	2 (20.0)	11 (68.8)	13 (50.0)	​
C	2 (20.0)	1 (6.3)	3 (11.5)	​
AFP >400 ng/mL, no. (%)	3 (30.0)	6 (37.5)	9 (34.6)	0.517
BCLC stage, no. (%)	​	​	​	0.426
A	1 (10.0)	0 (0)	1 (3.8)	​
B	0 (0)	0 (0)	0 (0)	​
C	7 (70.0)	13 (81.3)	20 (77.0)	​
D	2 (20.0)	3 (18.8)	5 (19.2)	​
Number of tumors, no. (%)	​	​	​	0.648
≤3	5 (50.0)	7 (43.8)	12 (46.2)	​
>3	5 (50.0)	9 (56.2)	14 (53.8)	​
Macroscopic vascular invasion, no. (%)	​	​	​	0.091
Yes	4 (40.0)	12 (75.0)	16 (61.5)	​
No	6 (60.0)	4 (25.0)	10 (38.5)	​
Extrahepatic metastasis, no. (%)	​	​	​	0.166
Yes	4 (40.0)	9 (56.3)	13 (50.0)	​
No	6 (60.0)	7 (43.7)	13 (50.0)	​
Hepatitis virus status, no. (%)	​	​	​	​
HBV infection	8 (80.0)	14 (87.5)	22 (84.6)	0.504
HCV infection	1 (10.0)	1 (6.3)	2 (7.7)	0.625
Previous treatment, no. (%)	​	​	​	​
Surgery	6 (60.0)	8 (50.0)	14 (53.8)	0.233
TACE or HAIC	6 (60.0)	12 (75.0)	18 (69.2)	​
Anti-cancer agent	2 (20.0)	13 (81.3)	15 (57.7)	​
EORTC QLQ-HCC18 score	​	​	​	​
Median (range)	20 (18–23)	21 (19–32)	21 (18–32)	0.674

Categorical data are presented as numbers (percentages), and quantitative data are presented as median values (interquartile ranges). Abbreviations: PS, performance status; TACE, transarterial chemoembolization; HAIC, hepatic arterial infusion chemotherapy; EORTC QLQ-HCC18, European Organization for Research and Treatment of Cancer Quality of Life Questionnaire for hepatocellular carcinoma.

### 3.2 Treatment response

Best treatment responses are summarized in Table 2. In the icaritin monotherapy group, there was one CR (10%) and five cases of SD (50.0%), yielding an ORR of 10% and a DCR of 60.0%. In the icaritin combination therapy group, there was one CR, five cases of PR (31.3%), and nine cases of SD (56.3%), resulting in an ORR of 37.5% and a DCR of 93.8%.

**TABLE 2:** Treatment response of patients to icaritin

Indicator	Icaritin monotherapy (N = 10)	Icaritin-based combination therapy (N = 16)
CR, no. (%)	1 (10.0)	1 (6.3)
PR, no. (%)	0 (0)	5 (31.3)
SD, no. (%)	5 (50.0)	9 (56.3)
PD, no. (%)	4 (40.0)	1 (6.3)
ORR (%)	10.0	37.5
DCR (%)	60.0	93.8

CR, complete response; PR, partial response; SD, stable disease; PD, progressive disease; ORR, objective response rate; DCR, disease control rate.

### 3.3 Survival profiles

**FIGURE 2:**
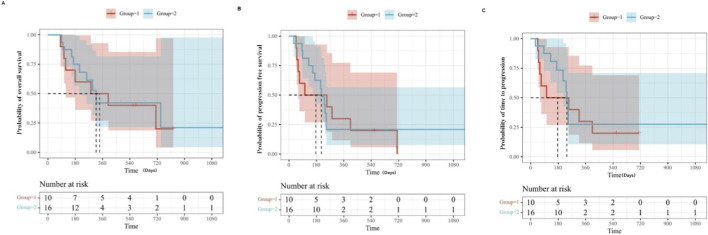
Prognosis of patients with icaritin treatment. The overall survival **(A)**, progression-free survival **(B)**, and time to progression **(C)** in the efficacious population are presented using Kaplan‒Meier curves. Group 1, monotherapy; Group 2, combination therapy.

The icaritin monotherapy group had a median PFS and TTP of 3.5 months (95% confidence interval (CI): 0.1–6.9) and a median OS of 9.4 months (95% CI: 0–20.7). The combination therapy group had a median PFS of 5.7 months (95% CI: 2.7–8.6), a median TTP of 7.1 months (95% CI: 2.9–11.3), and a median OS of 10.6 months (95% CI: 7.6–13.6). Overall, patients had a median PFS of 5.2 months (95% CI: 3.2–7.2), a median TTP of 4.7 months (95% CI: 1.9–7.5), and a median OS of 10.6 months (95% CI: 5.4–15.8) (Figure 2). Notably, one patient who was intolerant to both targeted and immunotherapy achieved a CR with icaritin monotherapy, with a PFS of 18 months. Detailed clinical characteristics of this case are provided in the case presentation section. Univariate Cox regression analysis indicated that age, sex, AFP score, liver function, prior therapies, and Hepatitis B virus (HBV) infection status were not independent prognostic factors for patient outcomes (Supplementary Table S1).

### 3.4 Safety

The most common TRAE in the monotherapy group was anemia (90.0%), whereas the combination group most frequently exhibited increased AST (93.8%), lymphopenia (93.8%), and anemia (93.8%). The overall incidence of grade 3 TRAEs was 13.9%. In the monotherapy group, the most common grade 3 TRAEs were elevated AST (20.0%) and elevated total bilirubin (TBIL) (20.0%). In the combination therapy group, grade 3 TRAEs included anemia (25.0%), increased TBIL (18.8%), hypoalbuminemia (18.8%), and lymphopenia (18.8%). No grade 4 or higher TRAEs or immune-related AEs were observed. As shown in Table 3, no treatment-related deaths or treatment discontinuations occurred due to TRAEs. There was no statistically significant difference in the incidence of TRAEs between the monotherapy group and the combination therapy group. Among five patients with BCLC stage D disease, hypoalbuminemia, anemia, and lymphopenia were observed; however, none of these events led to treatment interruption.

**TABLE 3:** Treatment-related adverse events in the monotherapy and combination therapy groups.

Adverse events	Icaritin monotherapy (N = 10)				Icaritin-based combination therapy (N = 15)				P value
	Grade 1	Grade 2	Grade 3	%	Grade 1	Grade 2	Grade 3	%	
Abdominal pain	4	0	0	40.0	3	1	0	26.7	0.636
Diarrhea	1	2	0	30.0	2	0	0	13.3	0.254
Abdominal distension	1	1	0	20.0	2	0	0	13.3	0.730
Nausea	0	1	0	10.0	2	0	0	13.3	0.302
Vomiting	0	1	1	10.0	1	0	0	6.7	0.650
ALT increased	2	0	1	30.0	2	0	0	13.3	0.409
AST increased	1	2	2	50.0	6	1	0	46.7	0.127
Hyperbilirubinemia	0	3	2	50.0	4	5	0	60.0	0.148
Alumin decreased	2	1	0	30.0	1	1	0	13.4	0.615
Hypophosphatemia	0	3	0	30.0	4	4	0	53.4	0.250
Proteinuria	2	0	0	20.0	2	2	1	33.3	0.776
Malaise	3	0	0	30.0	6	0	0	40.0	0.829
Leukopenia	0	0	0	0.0	2	2	0	16.0	0.391
Lymphopenia	2	3	0	50.0	7	10	1	72.0	0.242
Thrombocytopenia	2	1	0	37.5	3	2	2	53.8	0.903
Anemia	4	3	2	90.0	10	7	6	92.0	1.000

### 3.5 Subgroup and stratified analyses

Stratified analysis by icaritin dosage (high vs. low) revealed no statistically significant differences in OS or PFS between dose subgroups within either the monotherapy or combination therapy (Supplementary Figure S1). Additionally, a survival comparison between patients receiving combination targeted/immunotherapy and those receiving immunotherapy alone showed no significant differences in survival outcomes (Supplementary Figure S2).

A comparison of the incidence of TRAEs among the different combination regimens—targeted plus immunotherapy, immunotherapy alone, and targeted therapy alone—revealed a statistically significant difference (Supplementary Table S2). Overall, the subgroup receiving the combination of targeted and immunotherapy had the highest overall incidence.

### 3.6 Health-related quality of life

**FIGURE 3:**
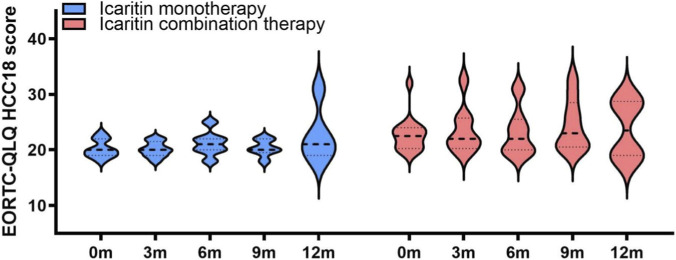
EORTC QLQ-HCC18 score of patients during icaritin treatment.

HRQOL remained stable or improved in the majority of patients in both groups (Figure 3). In the monotherapy group, 88.9% of patients maintained stable HRQOL at 3 months, with 11.1% showing improvement. By 12 months, 80.0% continued to exhibit stable HRQOL, while 20.0% showed improvement. In the combination therapy group, 75.0% had stable HRQOL, and 25.0% showed improvement at 3 months. By 12 months, 50.0% demonstrated improved HRQOL. The HRQOL scores at 3, 6, 9, and 12 months did not differ significantly from baseline in either group using a clinically substantial deterioration threshold of 10 points. These results indicate that HRQOL was generally maintained throughout the treatment period in both groups.

### 3.7 Case presentation

A 73-year-old male patient presented on 15 August 2022, with a 2-week history of abdominal pain and nausea. Contrast-enhanced CT revealed multiple intrahepatic lesions, with the largest measuring 11.5 cm in diameter, accompanied by tumor thrombus involving the inferior vena cava and right hepatic vein, as well as metastatic lymph nodes in the retroperitoneum and the right hemidiaphragmatic region. The patient also had HBV infection. Treatment was initiated with hepatic arterial infusion chemotherapy (HAIC) using oxaliplatin (180 mg) and raltitrexed (5 mg) on 19 August 2022. Immunotherapy with cardunolizumab (625 mg) began on 24 August 2022, but was permanently discontinued due to the occurrence of a severe generalized rash (CTCAE grade 4). A second cycle of HAIC was administered on 27 October 2022. From 31 October 2022, to 19 January 2023, the patient received lenvatinib at 8 mg daily; treatment was discontinued due to uncontrolled hypertension (CTCAE grade 4) and severe rash.

**FIGURE 4:**
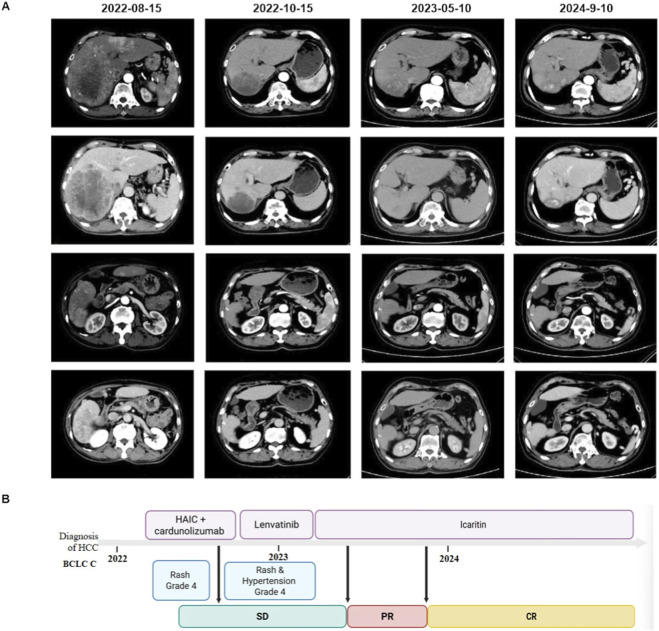
A representative patient treated with icaritin monotherapy. **(A)** CT images of the patient at initial diagnosis and during icaritin treatment. **(B)** Treatment process of patients.

In March 2023, follow-up imaging confirmed lung metastases. Icaritin monotherapy was initiated (300mg, BID) on 14 March 2023. After 2 months of treatment, the patient achieved a PR, which evolved into a CR by 14 November 2023. The most recent assessment on 10 September 2024, confirmed sustained CR, with PFS exceeding 18 months. Throughout treatment with icaritin, the patient experienced no grade ≥3 TRAEs and maintained stable HRQOL (Figure 4).

## 4 Discussion

This study retrospectively analyzed a case series of HCC patients treated with icaritin at our center, including both first-line and subsequent-line therapy settings. The efficacy and safety of icaritin as monotherapy, as well as in combination with targeted immunotherapy and TACE, were investigated. Our findings demonstrate that icaritin exhibits certain efficacy in HCC. Notably, icaritin was associated with a low incidence of adverse effects and exhibited favorable tolerability.

Despite advances in systemic therapies, managing HCC in real-world settings remains challenging, particularly for patients with impaired liver function, who are often excluded from first- and second-line regimens. In China, where HBV-related cirrhosis predominates, the majority of patients present with compromised liver function at diagnosis. This highlights an urgent need for safer therapies to address the unmet clinical needs of this specific population.

Icaritin has shown clinical potential for patients with advanced HCC and poor prognosis who are ineligible for or decline conventional therapies. Clinical studies demonstrated survival benefits, delayed progression, and minimal adverse events (Sun et al., 2021; Reyes-Hernández et al., 2024). A phase III trial reported a median OS of 13.54 months in an enriched population, comparable to the outcome achieved with sorafenib and lenvatinib, with only 12.1% of patients experiencing grade 3 or higher AEs (Sun et al., 2021). However, real-world evidence remains limited, warranting further investigation.

The majority of patients in our center’s icaritin monotherapy group were intolerant to targeted therapy, immunotherapy, or local therapy. Our data indicated that the median PFS for patients receiving icaritin monotherapy was 3.5 months, with a median OS of 9.4 months. Most patients receiving combination therapy with icaritin had developed resistance to multiple prior treatments. These patients often exhibited a poor prognosis (Ladd et al., 2024; Ntellas and Chau, 2024). In our study, combination therapy with icaritin resulted in a median OS of 10.6 months and a median PFS of 5.7 months. These findings suggest that icaritin demonstrated encouraging signals of activity that warrant prospective validation.

Previous studies indicate that HCC patients receiving best supportive care alone, without anti-tumor treatment, survive no more than 5 months (D'Avola et al., 2011). In a small retrospective cohort from our center, the median OS was just 1.5 months. Notably, in our study, 19.2% of patients had Child‒Pugh class C liver function or a performance status ≥2, rendering them ineligible for conventional therapies. The shortest OS was 2.8 months, and the median OS was 8.5 months. These findings suggest a potential survival benefit under the studied conditions, without compromising quality of life.

Stratification of patients by icaritinib dose showed no significant prognostic difference between the higher- and lower-dose groups. Similarly, subgroup analysis based on combined treatment regimens did not identify any significant survival differences among the groups. However, these findings should be interpreted cautiously, as the small sample size within each subgroup may have limited statistical power.

Icaritin shows protective effects on multiple organs, including the liver, lung, heart, bone, blood, and skin, and enhances immune responses (Huong and Son, 2023). Preclinical studies have suggested that icaritin provides analgesic benefits, potentially decreasing the degree of dependence on high-dose opioids (Yang et al., 2024). In our study, icaritin demonstrated a favorable safety profile, with grade 3 or higher TRAEs reported in 13.9% of patients, primarily involving liver function abnormalities and bone marrow suppression. This incidence is notably lower than those reported for targeted therapies (38%–57%) (Kudo et al., 2018; Qin et al., 2021), immunotherapy alone (22%) (Qin et al., 2023a), or combination therapies (35%–81%) (Ren et al., 2021; Qin et al., 2023b). Notably, no significant difference in TRAE rates was observed between the combination and monotherapy groups, implying that icaritin may attenuate the adverse effects associated with combination treatments. While combination therapy with both targeted and immunotherapies showed a higher AE incidence than single-agent combinations, this finding—based on limited sample sizes—should be interpreted cautiously. Nevertheless, it suggests that dual-targeted and immunotherapy combinations may warrant closer AE monitoring.

The liver represents a unique immunological organ in which antigen presentation is tightly regulated to balance immune surveillance with tolerance to gut-derived antigens. Hepatocytes, liver sinusoidal endothelial cells, Kupffer cells, and hepatic stellate cells all contribute to antigen processing and presentation through both major histocompatibility complex (MHC) class I and class II pathways. Once these strictly regulated networks are disrupted, the balance shifts in a pathological direction, leading not only to tumor immune escape but also to immune-mediated liver damage such as autoimmune hepatitis (AIH) and ultimately promoting tumorigenesis (Aravalli, 2013; Mossanen and Tacke, 2013; Fasano et al., 2021). Icaritin has been reported to regulate immune cell activation and function, modulate inflammatory factor release, and restore dysregulated signaling pathways (Bi et al. 2022). Notably, icaritin has been shown to reduce the generation and activation of myeloid-derived suppressor cells and was found to synergistically enhance the efficacy of ICIs in HCC mouse models (Tao et al., 2021). Icaritin has also been demonstrated to exert hepatoprotective effects by attenuating inflammation, oxidative stress, and excessive autophagy, thereby alleviating ischemia–reperfusion (IR) injury (Sun et al., 2024). We suspect that icaritin may rebalance antigen presentation pathways toward a state of controlled immunity that could mitigate the heightened risk of immune-mediated injury in damaged livers without exacerbating immunosuppression, thereby offering a therapeutic profile aligned with the liver’s intrinsic immunological architecture.

Maintaining or improving the quality of life is a critical aspect of HCC treatment. Previous clinical studies reported that the TTD for targeted monotherapy was approximately 3–4 months (Kudo et al., 2018; Llovet et al., 2008) and that for combination therapy was approximately 1 month (Cheng et al., 2022; Yang et al., 2024; Llovet et al., 2023) (Supplementary Table S3). In the present study, patients treated with icaritin, either as monotherapy or in combination therapy, maintained a stable quality of life over a median follow-up of 10.6 months, with no reported deterioration in quality of life.

Several limitations exist in this study. First, patients receiving icaritin were those intolerant to or ineligible for standard treatment. This population constitutes a small proportion in clinical practice, resulting in a limited sample size in the present study, which may restrict the generalizability of the findings. Second, the retrospective, lack of randomization design introduces potential biases, including selection bias and incomplete data collection. Third, the lack of a control group precludes direct comparisons with standard systemic therapies. In the future, large-scale, multicenter real-world cohort studies employing propensity score matching and advanced statistical methods will be essential to robustly validate these findings across diverse populations and settings.

## 5 Conclusion

In conclusion, this study suggested that icaritin, either as monotherapy or in combination with other therapies, demonstrates potential efficacy and a favorable safety profile for patients with uHCC. Combination therapies showed potential clinical activity in improving patient prognosis. These findings support the potential use of icaritin as a therapeutic option for patients who are ineligible for standard therapy. Future multicenter, prospective studies with larger cohorts will be conducted to further validate the efficacy and safety of icaritin in this patient population. Additionally, efforts are needed to identify and validate predictive biomarkers that may guide patient selection for icaritin-based therapies.

## Data Availability

The raw data supporting the conclusions of this article will be made available by the authors, without undue reservation.
