# Supplementary material for: Clinical efficacy and safety of icaritin in patients with hepatocellular carcinoma: a real-world study
Source: Front Pharmacol. 2026 Feb 12;17:1677794. doi: 10.3389/fphar.2026.1677794 (PMC12935929; doi:10.3389/fphar.2026.1677794)

**Supplementary Table S1**. Results of univariate Cox regression analysis of the factors that might affect the prognosis of the patients.

| **Factors** | **P value** |
| --- | --- |
| Gender | 0.46 |
| Age | 0.074 |
| Liver function | 0.56 |
| AFP | 0.094 |
| HBV infection status | 0.087 |
| Prior therapy | 0.83 |

**Supplementary Table S2**. Comparison of adverse reactions among different combined treatment strategies.

|  |  |  |  |  |  | |  |  |  |
| --- | --- | --- | --- | --- | --- | --- | --- | --- | --- |
| **Adverse Events** | **Combined immunotherapy (n=3**) |  | **Combined targeted therapy (n=2)** | | | **Combined targeted + immunotherapy (n=9)** | |  | **P value** |
|  | **No.** | **%** |  | **No.** | **%** | | **No.** | **%** |  |
| Abdominal pain | 1 | 33.3 |  | 0 | 0.0 | | 4 | 44.4 | <0.001 |
| Diarrhea | 0 | 0.0 |  | 0 | 0.0 | | 1 | 11.1 | <0.001 |
| Abdominal distension | 1 | 33.3 |  | 0 | 0.0 | | 1 | 11.1 | <0.001 |
| Nausea | 1 | 33.3 |  | 0 | 0.0 | | 2 | 11.1 | <0.001 |
| Vomiting | 0 | 0.0 |  | 0 | 0.0 | | 1 | 0.0 | <0.001 |
| ALT increased | 1 | 33.3 |  | 1 | 50.0 | | 1 | 11.1 | <0.001 |
| AST increased | 2 | 66.7 |  | 1 | 50.0 | | 4 | 44.4 | <0.001 |
| Hyperbilirubinemia | 2 | 66.7 |  | 1 | 50.0 | | 7 | 77.8 | <0.001 |
| Alumin decreased | 2 | 66.7 |  | 1 | 50.0 | | 7 | 77.8 | <0.001 |
| Hypophosphatemia | 0 | 0.0 |  | 1 | 50.0 | | 6 | 66.7 | <0.001 |
| Proteinuria | 2 | 66.7 |  | 2 | 100.0 | | 8 | 88.9 | <0.001 |
| Malaise | 1 | 33.3 |  | 2 | 100.0 | | 3 | 33.3 | <0.001 |
| Leukopenia | 1 | 33.3 |  | 0 | 0.0 | | 4 | 44.4 | <0.001 |
| Lymphopenia | 3 | 100.0 |  | 1 | 50.0 | | 9 | 100.0 | <0.001 |
| Thrombocytopenia | 2 | 66.7 |  | 1 | 50.0 | | 7 | 77.8 | <0.001 |
| Anemia | 3 | 100 |  | 2 | 100 | | 9 | 100 | / |
|  |  |  |  |  |  | |  |  |  |

**Supplementary Table S3**. Health-related quality of life outcomes in HCC trials.

| **Trial** | **Treatment arms** | **Questionnaire version** | **TTD (months)** |
| --- | --- | --- | --- |
| sharp | sorafenib vs placebo | FHSI8 | 4.1 vs 4.9 |
| reflect | lenvatinib vs sorafenib | EORTC QLQ-C30  EORTC QLQ-HCC18 | not significantly different |
| IMbrave150 | A+T vs sorafenib | EORTC QLQ-C30 | 11.2 vs. 3.6  (HR 0.63，95%CI 0.46-0.85) |
| LEAP002 | Pembrolizumab + lenvatinib vs lenvatinib | EORTC QLQ-C30  EORTC QLQ-HCC18 | 11.5 vs. 4.3 |
| CARES-310 | Camrelizumab + rivoceranib vs sorafenib | EORTC QLQ-C30 | 11.2 vs. NR  (HR 1.02, 95% CI 0.77-1.36) |

Note: TTD, time to deterioration

**Supplementary Figure S1**. Survival analysis of patients with low versus high icaritin treatment in the monotherapy and combination therapy groups.


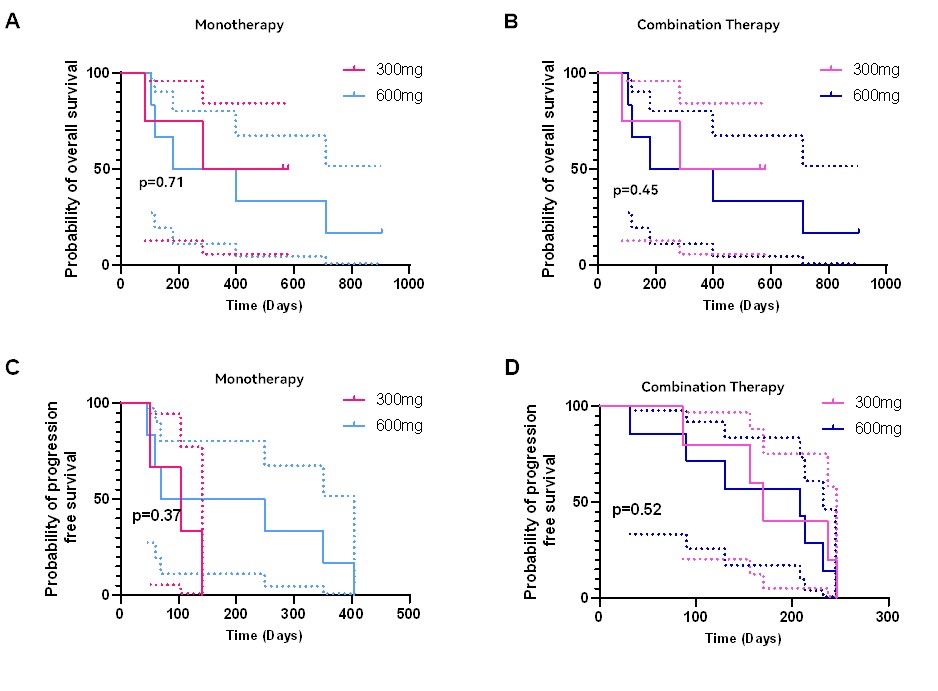


**Supplementary Figure S2.** Survival analysis of patients in the combination group receiving targeted-immunotherapy vs. immunotherapy alone


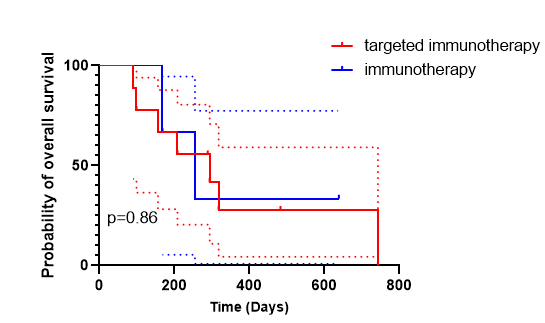

Supplement: Supplementary file 1 [file DataSheet1.docx]
